# Obesity, metabolic syndrome, impaired fasting glucose, and microvascular dysfunction: a principal component analysis approach

**DOI:** 10.1186/1471-2261-12-102

**Published:** 2012-11-13

**Authors:** Diogo G Panazzolo, Fernando L Sicuro, Ruth Clapauch, Priscila A Maranhão, Eliete Bouskela, Luiz G Kraemer-Aguiar

**Affiliations:** 1 Clinical and Experimental Research Laboratory on Vascular Biology (BioVasc),Biomedical Center, State University of Rio de Janeiro, Rio de Janeiro, Brazil; 2 Female Endocrinology Sector, Hospital da Lagoa, Health Ministry, Rio deJaneiro, Brazil; 3 Division of Endocrinology, Department of Internal Medicine, Medical Sciences Faculty, State University of Rio de Janeiro, Rio de Janeiro, Brazil

**Keywords:** Microcirculation, Capillaries, Obesity, Metabolic syndrome, Impaired fasting glucose, Principal component analysis

## Abstract

**Background:**

We aimed to evaluate the multivariate association between functional microvascular variables and clinical-laboratorial-anthropometrical measurements.

**Methods:**

Data from 189 female subjects (34.0±15.5 years, 30.5±7.1 kg/m^2^), who were non-smokers, non-regular drug users, without a history of diabetes and/or hypertension, were analyzed by principal component analysis (PCA). PCA is a classical multivariate exploratory tool because it highlights common variation between variables allowing inferences about possible biological meaning of associations between them, without pre-establishing cause-effect relationships. In total, 15 variables were used for PCA: body mass index (BMI), waist circumference, systolic and diastolic blood pressure (BP), fasting plasma glucose, levels of total cholesterol, high-density lipoprotein cholesterol (HDL-c), low-density lipoprotein cholesterol (LDL-c), triglycerides (TG), insulin, C-reactive protein (CRP), and functional microvascular variables measured by nailfold videocapillaroscopy. Nailfold videocapillaroscopy was used for direct visualization of nutritive capillaries, assessing functional capillary density, red blood cell velocity (RBCV) at rest and peak after 1 min of arterial occlusion (RBCV_max_), and the time taken to reach RBCV_max_ (TRBCV_max_).

**Results:**

A total of 35% of subjects had metabolic syndrome, 77% were overweight/obese, and 9.5% had impaired fasting glucose. PCA was able to recognize that functional microvascular variables and clinical-laboratorial-anthropometrical measurements had a similar variation. The first five principal components explained most of the intrinsic variation of the data. For example, principal component 1 was associated with BMI, waist circumference, systolic BP, diastolic BP, insulin, TG, CRP, and TRBCV_max_ varying in the same way. Principal component 1 also showed a strong association among HDL-c, RBCV, and RBCV_max_, but in the opposite way. Principal component 3 was associated only with microvascular variables in the same way (functional capillary density, RBCV and RBCV_max_). Fasting plasma glucose appeared to be related to principal component 4 and did not show any association with microvascular reactivity.

**Conclusions:**

In non-diabetic female subjects, a multivariate scenario of associations between classic clinical variables strictly related to obesity and metabolic syndrome suggests a significant relationship between these diseases and microvascular reactivity.

## Background

The microcirculation, represented by arterioles, capillaries and venules, is where blood/tissue nutrition and exchange effectively takes place. Several techniques have been proposed and used to assess microvascular reactivity in the microcirculation. Among these techniques, nailfold videocapillaroscopy is a non-invasive technique that actually visualizes capillaries, assessing skin nutritive microvascular flow and reactivity.

The relationship between blood glucose and the microcirculation requires further clarification, because even in non-diabetics, insulin resistance is a significant predictor of poor outcome in patients admitted with myocardial infarction [1,2]. Additionally, many studies of small sample sizes have documented that skin capillary recruitment, an index of healthy tissue status, is related to insulin resistance and blood pressure (BP), even in normotensive [3] and obese subjects [4,5]. Microvascular dysfunction has also been observed in non-diabetic metabolic syndrome patients [6] and in non-diabetic obese women independently of metabolic syndrome diagnosis [7]. The concept of microvascular dysfunction as the pathophysiological basis of a pre-receptor defect aggravating insulin resistance and its cause-effect relationship is still a matter of debate [8].

The endothelial glycocalyx is a network of membrane-bound proteoglycans and glycoproteins covering the endothelium in the luminal side. Both endothelium and plasma-derived soluble molecules integrate into this mesh. In healthy vessels glycocalyx determines vascular permeability, attenuates blood cell–vessel wall interactions, mediates shear stress sensing, enables balanced signaling, and fulfills a vasculoprotective role. However, in states of disease, experimental settings [9,10] have suggested that the altered glycocalyx homeostasis affects endothelial function. Endothelial dysfunction is considered a precocious marker of atherosclerotic risk [11,12]. Moreover, because of the systemic nature of such dysfunction, which can simultaneously affect the coronary circulation as well as peripheral vascular beds, it has emerged that endothelial dysfunction in peripheral conduit arteries, small resistance vessels, and skin nutritive microcirculation can be used as a surrogate marker of coronary endothelial/microvascular damage [13,14].

Microvascular reactivity occurs at the level of small pre-capillary arterioles, which are considered as the main regulators of capillary reactivity, also contributing to total peripheral vascular resistance. Nailfold videocapillaroscopy is used to measure capillary variables that reflect endothelial control at pre-capillary sites. In metabolic diseases, correlations between findings with dynamic videocapillaroscopy and observed findings in target organs still need further elucidation. We have previously shown, that in response to an insulin-sensitizing agent given to metabolic syndrome normoglycemic patients, there was a concomitant improvement in skin microvascular dysfunction [15] and in endothelial microvascular reactivity in the muscle [16]. Notably, the drug that we used did not affect endothelial-independent vasodilation, suggesting an endothelial-dependent mechanism controlling capillary parameters, as observed by nailfold videocapillaroscopy. At microvascular sites during a reactive hyperemia response, the role of reactive oxygen species secondary to hypoxia could also influence microvascular reactivity.

The current study investigated a large number of patients, tested by dynamic nailfold videocapillaroscopy, in a sample of predominantly non-diabetic non-hypertensive obese female subjects, analyzed by principal component analysis. PCA is a multivariate exploratory approach used to identify common variation between analyzed variables, aiming to reduce the dimensionality of the data set and to detect the main source of inherent variation among investigated variables. It should be noted that the main assumption for this statistical method does not pre-establish any possible cause-effect relationship between variables. By grouping variables that behave in a similar fashion, they form a principal component, and by doing so, the researcher can suggest a biological phenomenon associated with it, and even name the principal component. Our study aimed to explore trends and associations between microvascular function and classic clinical measurements frequently used in a clinical cardiometabolic setting without pre-establishing cause-effect phenomena.

## Methods

### Study population

The present study included data from research protocols of the Clinical and Experimental Research Laboratory on Vascular Biology (BioVasc), from November 2005 to May 2010, located in Rio de Janeiro, RJ, Brazil. All selected protocols were composed of only female subjects, because of the small number of male subjects in our database. Inclusion criteria were female subjects, non-regular drinkers, and nonsmokers. To avoid drug bias on microvascular function, no subject who was regularly using any drug, including oral contraceptives and aspirin, and anti-hyperlipidemic, anti-hypertensive, or anti-hyperglycemic agents, had their data entered in the database. Female adolescents were included only at Tanner stage ≥ 4 [17]. Exclusion criteria were pregnancy, prepubertal status, known history of type 2 diabetes and/or hypertension, a history of previous myocardial infarction or angina pectoris, hypertriglyceridemia (≥4.52 mmol/l), and systemic diseases, such as autoimmune diseases (rheumatologic or thyroid diseases), cancer, or active infection. In total, data from 189 subjects (34.0±15.5 years, 30.5±7.1 kg/m^2^) were selected from a large age range group, 12–64 years, and classified according to body mass index (BMI) as normal weight, overweight, or obese [18,19]. They were also categorized as subjects with or without metabolic syndrome according to the International Diabetes Federation [20] or to the Joint Interim Statement [21], respectively, for those up to 16 years or older than 16 years. Hypertension was categorized according to The Seventh Report of the Joint National Committee on Prevention, Detection, Evaluation, and Treatment of High Blood Pressure (JNC 7) [22].

Study protocols were approved by the Ethical Committees of the Hospital da Lagoa (02/2005) and State University of Rio de Janeiro (COEP1950/2007). Written informed consent was obtained for all subjects, while for subjects <18 years old, a parent or guardian gave the consent and signed it.

### Data collection

#### Anthropometric, blood pressure, and laboratory measurements

Anthropometric, blood pressure (BP), and laboratory measurements were well-validated as previously reported [4,6,7,15,23]. The body weight of subjects wearing light clothing without shoes was measured with a 0.1-kg precision, height was measured to the nearest 0.5 cm, and BMI was calculated as the weight in kilograms divided by the square of height in meters. Waist circumference was defined as the average of two measurements taken after inspiration and expiration at the midpoint between the lowest rib and the iliac crest. BP measurements were made twice using the appropriate cuff size after the subject had rested for 5 min in the sitting position using a standard sphygmomanometer. All laboratory measurements were performed in duplicate after 10–12 h of fasting using an automated method (Modular Analytics E 170 and P, Roche, Basel, Switzerland). Fasting plasma glucose, total cholesterol, triglycerides (TG), and high-density lipoprotein cholesterol (HDL-c) levels were measured by the enzyme-colorimetric oxidase-peroxidase method (inter-assay coefficient of variation [IECV]=1.09%), the enzymatic oxidase-peroxidase method (IECV=2.93%), the enzymatic oxidase-peroxidase method (IECV=1.29%), and the enzyme-colorimetric method without pre-treatment (IECV=3.23%), respectively. Plasma low-density lipoprotein cholesterol (LDL-c) levels were calculated according to the Friedewald equation. C-reactive protein (CRP) levels were measured by immunoturbidimetry (IECV=8%). Serum insulin levels were analyzed by eletrochemiluminescence (IECV=10.6%). Subjects with fasting plasma glucose levels ≥ 5.6 mmol/l were subsequently given an oral glucose tolerance test according to the American Diabetes Association criteria [24].

#### Microvascular assessment

Nailfold videocapillaroscopy was carried out and analyzed according to a standardized, well-validated methodology, as previously described [4,6,7,25], for the 4^th^ finger of the left hand after a 10**–**12-h fast. All women were acclimatized for 30 min in a room kept at 24 ± 1°C before conducting the examination. Whether the menstrual cycle phase affects microvascular reactivity is still controversial [26,27], but we assessed skin microcirculation without adjustment for the menstrual cycle because it has previously been shown that the skin microcirculation is not affected by menstrual phases [27]. Measurements of microvascular reactivity were made by a Leica DMLM microscope (Wetzlar, Germany), which was equipped with an epiillumination system (100 W Xenon lamp). Images were captured by a CCD video camera (Samsung, Seoul, South Korea) coupled to the microscope, visualized by a Kodo KBM1700E monitor (Seoul, South Korea) and recorded by a super VHS videotape recorder (Super VHSET, JVC, Malaysia). The subject’s fingertip was fixed to the acrylic base by a metal loop to minimize movement. The skin temperature of the finger was monitored throughout the examination with an YSI Precision 4000A digital thermometer (Dayton, OH, U.S.A.) with the thermistor probe taped within 1 cm proximal of the nailfold. A pressure cuff (1 cm wide) was placed around the proximal phalanx of the 4^th^ finger and connected to a mercury manometer. The exam was continuously recorded for later measurements of microvascular variables using CapImage software [28] by the same observer, who was not aware of any patient data. With the patient at rest, functional capillary density, which is the number of capillaries/unit tissue area (mm^2^) with flowing red blood cells, was evaluated using x250 magnification and an area of 3 mm of the distal row of capillaries into three different areas (intra-assay coefficient of variation [CV] = 5.5±2.5%). Red blood cell velocity (RBCV) at rest and its peak after 1 min of arterial occlusion (RBCV_max_), which was achieved by a pressure cuff placed around the proximal phalanx, and the time taken to reach RBCV_max_ (TRBCV_max_) were measured with a final magnification of x680, before and during the post-occlusive reactive hyperemia response. Conceptually, functional capillary density and RBCV tested at rest, and RBCV_max_ and TRBCV_max_ assessed during post-occlusive reactive hyperemia, are considered functional variables. Time resolution for velocity measurements for the non-interlaced 30 video frames per second (fps) used, corrected for image persistence, is 0.2 s. Nailfold videocapillaroscopy is a well-validated method, and our intra-assay coefficients of variation have been previously reported [7,25].

#### Statistical analysis

We used StatSoft, 2004 software (STATISTICA, version 7; Tulsa, OK, USA) for data analysis, and variables were tested regarding their problems of distribution (i.e., normality, kurtosis, skewness, and homoscedasticity). BMI, waist circumference, systolic BP, diastolic BP, levels of fasting plasma glucose, insulin, total cholesterol, HDL-c, LDL-c, TG, and CRP, and functional capillary density, RBCV, RBCV_max_, and TRBCV_max_ were all Z-standardized and subsequently analyzed. PCA is a classical multivariate statistical technique that was first described by Pearson in 1901 [29], and after the advent of the electronic computers, it became one of the more usual multivariate data mining approaches[30]. The main aim of this method is to detect the common variation between original variables, and then condense a large dataset into a few derived variables, named as principal components (PCs). The obtained principal components are linear combinations of original variables with some degree of correlation between them. The different principal components, however, are – by definition – uncorrelated among themselves. Therefore, the first component obtained in this analysis accounts for a maximal amount of total variation between variables, while the following component will account for the maximal amount of variance not accounted for by the precedent one. This means that the second component will be more correlated with some of the observed variables that did not display strong correlations with the first one, and therefore, it will be uncorrelated with the first component. These assumptions are the same for the remaining components that are extracted in the analysis, meaning that each new component will progressively account for smaller and smaller amounts of variance, expressed by “eigenvalues”. The eigenvalue is the sum of squared correlations between original independent variables and the principal components obtained, and it represents the amount of variance attributable to each component. We used the Kaiser-Guttman method for principal components selection (i.e., eigenvector Lambda’s >1, and the plot of eigenvalues according to components [scree-plot] as indicative of the relevance of the principal component for interpretation of the data) [31,32]. The degree of correlation between variables and principal components are given by variable loadings. Therefore, the higher the loading value is, the higher the influence of a given variable on a principal component. Based on variables with higher loadings, the biological meaning of the principal components can be interpreted. In our study, the cutoff for variable loadings was arbitrarily established as ≥0.45, and those with higher values were considered as main contributor(s) to each principal component and used to define meanings (principal component labels) [33].

Since the principal components are derived variables, each individual included in the experiment has a score related to each principal component. Based on these scores, each subject was grouped *a posteriori* according to BMI classes and metabolic syndrome diagnosis, and then differences between these groups were tested by analysis of variance (ANOVA). P values ≤0.05 were considered statistically significant.

## Results

Table 1 depicts anthropometrical, clinical-laboratorial, and functional microvascular variables of 189 female subjects. Thirty-five percent (n=66) of the subjects had metabolic syndrome and 77% (n=146) were overweight. None of the subjects were at the pre-pubertal stage, 67% (n=127) were of fertile age, and 33% (n=62) were in the postmenopausal period. Metabolic syndrome was diagnosed according to age as follows: 36% (n=13) of subjects were ≤18 years (19% [n=36]); 32% (n=22) of subjects were between 19**–**30 years (36% [n=68]); 50% (n=20) of subjects were between 31–50 years (21% [n=40]); and 24% (n=11) of subjects were ≥51 years (24% [n=45]). Fifty-nine subjects (31.2%) were normotensive, 89 (47.1%) had prehypertension, 35 (18.5%) were at stage 1 hypertension, and 6 (3.2%) were at stage 2 but without ongoing treatment. All subjects with ≥JNC 7 stage 1 were referred to the outpatient care unit for treatment. Eighteen (9.5%) subjects had impaired fasting glucose, but all of them had a 2-h post-load glucose below 7.8 mmol/l.

**Table 1:** Anthropometrical, clinical-laboratorial, and functional microvascular variables of 189 female subjects

**Variables**	**Mean**±**SD / Median [1**^**st**^**-3**^**rd**^**]**	**(Min-Max)**
Age (years)	29 [20.5-49.5]	(12–64)
BMI (kg/m^2^)	30.5±7.1	(16.2-56.4)
Waist circumference (cm)	92.6±15.72	(60–146)
Systolic blood pressure (mmHg)	125.1±16	(80–182)
Diastolic blood pressure(mmHg)	74.8±10	(50–123)
Fasting plasma glucose (mmol/l)	4.86±0.55	(3.27-6.94)
Insulin (pmol/l)	76.06 [45.6-123.7]	(13.1-333.3)
Total cholesterol (mmol/l)	4.86±1.09	(1.68-8.10)
Triglycerides (mmol/l)	1.33±0.66	(0.30-3.83)
HDL cholesterol (mmol/l)	1.33±0.39	(0.46-2.53)
LDL cholesterol (mmol/l)	2.92±0.93	(0.73-5.82)
C-reactive protein (mg/l)	3.00 [1.15-6.85]	(0.3-20)
FCD (cap/mm^2^)	10.0 [7.6-14.4]	(4.3-29.16)
RBCV (mm/s)	0.30±0.08	(0.07-0.41)
RBCV_max_ (mm/s)	0.36±0.05	(0.12-0.48)
TRBCV_max_ (s)	8.37±2.29	(3–15)

FCD, functional capillary density; RBCV, baseline red blood cell velocity; RBCV_max_, peak red blood cell velocity; TRBCV_max_, time taken to reach RBCV_max_ during the reactive hyperemia response.

According to the Kaiser-Guttman method [31], only the first five principal components had eigenvalues ≥ 1.0 and accounted for 72.5% of the total variation. Loadings of more related variables to each principal component are presented in Table 2. For principal component 1, which explained 29% of the variation, 11 variables presented loads higher than the established cutoffs, showing a strong relationship among BMI, waist circumference, systolic and diastolic BP, insulin levels, TG levels, CRP levels, and TRBCV_max_ in the same way of variation, and also a strong association among HDL-c, RBCV, RBCV_max_, but in the opposite way. For principal component 2, two variables (total cholesterol and LDL-c levels) reached the cutoff value, accounting for 16.5% of the variation. Explaining 12% of the total variation, only microvascular variables (functional capillary density, RBCV and RBCV_max_) loaded principal component 3. Unexpectedly, fasting plasma glucose appeared for the first time only in principal component 4, contributing to 8% of the variation, but without an association with any microvascular variable. For principal component 5, which explained 7% of the variation, only diastolic BP presented with a load higher than the established cutoff. One of the assumptions of this statistical method allows the designation of principal components according to what they appear to express from a biological view. Principal component 1 could be designated as the abdominal obesity and insulin resistance component, clinically expressed as metabolic syndrome, with principal component 2 as the dyslipidemic component. Principal component 3 could be designated as the microvascular component, while principal components 4 and 5 could be viewed as glycemic and pressure components, respectively. All of the analyses described above were performed excluding subjects ≤18 years. RBCV_max_ loading on principal component 3 lost its significance, but any other significant difference was not observed (data not shown).

**Table 2:** Variable loadings^**a **^related to each principal component

	**Principal Components**				
	**PC1**	**PC2**	**PC3**	**PC4**	**PC5**
BMI	**0,80**	0,20	−0,23	0,20	−0,28
Waist circumference	**0,75**	0,23	−0,39	0,18	−0,15
Systolic blood pressure	**0,72**	−0,21	0,03	0,39	0,38
Diastolic blood pressure	**0,56**	−0,38	0,03	0,35	**0,54**
Fasting plasma glucose	0,19	−0,15	−0,31	**−0,59**	0,36
Insulin	**0,62**	0,35	−0,32	−0,17	−0,11
Total cholesterol	−0,03	**−0,95**	0,00	−0,04	−0,23
Triglycerides	**0,45**	−0,42	−0,20	−0,44	−0,07
HDL cholesterol	**−0,55**	−0,24	0,41	0,20	−0,09
LDL cholesterol	0,05	**−0,88**	−0,12	0,02	−0,22
C-reactive protein	**0,50**	−0,05	0,23	0,30	−0,37
FCD	−0,29	−0,13	**−0,63**	0,14	−0,22
RBCV	**−0,58**	0,01	**−0,65**	0,24	0,10
RBCV_max_	**−0,67**	−0,15	**−0,51**	0,27	0,14
TRBCV_max_	**0,52**	−0,36	0,01	−0,06	0,05
Proportion of total variation	29%	16.5%	12%	8%	7%

^a^Loading denotes the contribution of the variable to each PC. Higher absolute value indicates that the variable has a major influence on the PCs. Bolded numbers indicate loadings ≥ 0.45.

PC, principal component; FCD, functional capillary density; RBCV, baseline red blood cell velocity; RBCV_max_, peak red blood cell velocity; TRBCV_max_, time taken to reach RBCV_max_ during the reactive hyperemia response.

Using BMI as a grouping variable, we categorized our subjects as normal weight, overweight, and obese, and observed significant differences in four principal components (PC1: *p*<0.001; PC2: *p*<0.01; PC3: *p*<0.01; PC5: *p*<0.01). According to their principal component 1 scores, chosen BMI classes could be differentiated (Tukey’s HSD, *p*<0.001), where normal weight subjects were more related to HDL-c, RBCV, and RBCV_max_, while obese subjects were more related to higher values of variables that were composed of principal component 1 (BMI, waist circumference, systolic BP, diastolic BP, insulin levels, TG levels, CRP levels, and TRBCV_max_). Analysis of principal component 3 scores according to BMI classes indicated significant differences between normal weight and overweight (Tukey’s HSD, *p*<0.03), and also between normal weight and obesity (Tukey’s HSD, *p*<0.01), with no difference between subjects who were overweight and those who were obese. Normal weight subjects showed an association with higher values of functional capillary density, RBCV and RBCV_max_.

Principal component 1 was the only component able to show differences between subjects with and those without metabolic syndrome (PC1: *p*<0.001), and this diagnosis was associated with higher values of variables intrinsically related to insulin resistance but also to a prolonged microvascular reactive hyperemia response, such as BMI, waist circumference, systolic BP, diastolic BP, insulin, TG, CRP and TRBCV_max_).

## Discussion

Microvascular damage is a well-known complication of type 2 diabetes, but data on microvascular damage in non-diabetic obese subjects are still scarce. However, there are some studies that have described a possible etiopathogenetic relationship between states of metabolic disorders without diabetes and microvascular derangement [4,7]. We previously observed microvascular dysfunction in normoglycemic metabolic syndrome subjects [6], and also a possible effect of waist circumference on microvascular reactive hyperemia responses [7]. In the current study, although some subjects were diagnosed with hypertension and impaired fasting glucose, at microvascular assessment, none of them were using any drug that could result in a drug bias for the analyzed data. Consistent with our previously published data [34], we observed that, although 9.5% of the subjects had impaired fasting glucose, these levels of glycemia were not associated with any functional microvascular variables in our exploratory analysis. These findings suggest an etiopathogenetic relationship between microvascular dysfunction and excessive adiposity, without establishing a cause-effect relationship, but possibly antedating type 2 diabetes diagnosis. When comparing subjects by BMI classes, our data supported that the level of adiposity could be directly associated with microvascular dysfunction, in which a higher BMI was related to an impaired microvascular reactivity, which is corroborated by previous findings even in the absence of conditions, such as hypertension [35], hypercholesterolemia [36], and hyperglycemia [37]. These findings suggest that obesity and metabolic syndrome are related to microvascular dysfunction.

Microvascular variables associated with clinical variables were present in two principal components, 1 and 3, with the latter expressing exclusively the microcirculation. For principal component 1, which explained most of the variance, RBCV and RBCV_max_ were both associated with HDL-c levels on the same way, suggesting that this lipoprotein could play a protective role in the microcirculation. Additionally, obesity, mainly abdominal adiposity, as expressed by BMI and waist circumference, were the main factors (higher factor loadings) associated with the time taken to reach peak red blood cell velocity (TRBCV_max_) during the reactive hyperemia response. Prolongation of the reactive hyperemia response was also associated with increased blood pressure, insulinemia, and CRP levels in principal component 1, reassuring our previous findings in other small samples [4,6]. These data strongly suggest that other mechanisms related to abdominal obesity rather than hyperglycemia are involved in the observed prolonged time for the microvascular reactive hyperemia response in early stages of metabolic diseases, and together microcirculation and precocious metabolic derangement as part of the same pathophysiological process without establishing an exact cause-effect relationship.

In a nondiabetic lean/overweight population, Voidonikola and co-workers [38] reported that long-term glycemia levels are associated with endothelial dysfunction only in lean individuals, while in overweight individuals, this association is not apparent. Similarly, Han and co-workers [39] reported that dysglycemia contributes to impaired vascular function in non-obese subjects, but in obese and diabetic subjects, obesity and insulin resistance *per se* are more important determinants of vascular function than dysglycemia. These recent studies examining macrovascular reactivity support and parallel our findings on nutritive microcirculation because our population was predominantly composed of obese female subjects with insulin resistance but without hyperglycemia.

Our study has some limitations. Sex specificities and their effect on vascular homeostasis should be considered. The addition of male subjects might reinforce our results, because the protective aspect of the female hormonal milieu is well-established in the cardiovascular literature, and our group was predominantly composed of fertile female subjects. The large age range in the investigated group led to differences in the prevalence of obesity/metabolic syndrome and hormonal status, which could also be viewed as a possible bias for the analysis, but the proposed statistical procedure reduced this age bias.

## Conclusions

In conclusion, based on our PCA, we found that the nutritive microcirculation of non-diabetic female subjects with a wide age range and with different adiposity levels and metabolic impairment, is associated with classic clinical variables, but not with fasting plasma glucose levels. Therefore, we postulate that, in our cross-sectional study population, impaired fasting glucose is not associated with precocious microvascular dysfunction, and obesity and metabolic syndrome *per se* appear to be more important for observed relationships.

## Abbreviations

BP: Blood pressure; CRP: C-reactive protein; HDL-c: High-density lipoprotein cholesterol; LDL-c: Low-density lipoprotein cholesterol; PCA: Principal component analysis; RBCV: Red blood cell velocity; RBCV_max_: Peak red blood cell velocity; TG: Triglycerides; TRBCV_max_: Time taken to reach RBCV_max_.

## Competing interests

The authors declare that they have no competing interests.

## Authors’ contributions

DGP wrote the manuscript, analyzed the data and contributed to the discussion. RC collected the data. PAM collected the data. FLS wrote the manuscript, analyzed the data, and contributed to the discussion. EB reviewed/edited the manuscript. LGKA wrote the manuscript, analyzed the data, contributed to the discussion, and reviewed/edited the manuscript. All authors read and approved the final manuscript.

## Pre-publication history

The pre-publication history for this paper can be accessed here:

http://www.biomedcentral.com/1471-2261/12/102/prepub
